# Unraveling the Intricacies of CD73/Adenosine Signaling: The Pulmonary Immune and Stromal Microenvironment in Lung Cancer

**DOI:** 10.3390/cancers15235706

**Published:** 2023-12-04

**Authors:** Maria Saigí, Oscar Mesía-Carbonell, David A. Barbie, Raquel Guillamat-Prats

**Affiliations:** 1 Badalona-Applied Research Group in Oncology (B-ARGO), Department of Medical Oncology, Catalan Institute of Oncology (ICO), Germans Trias i Pujol Research Institute (IGTP), Carretera de Canyet, s/n, 08916 Badalona, Spain; 2 Lung Immunity Translational Research Group in Respiratory Diseases, Germans Trias i Pujol Research Institute (IGTP), 08914 Badalona, Spain; 3 Facultad de Biomedicina, Universitat de Barcelona (UB), 08007 Barcelona, Spain; 4 Department of Medical Oncology, Dana-Farber Cancer Institute, Boston, MA 02115, USA; 5 Belfer Center for Applied Cancer Science, Dana-Farber Cancer Institute, Boston, MA 02115, USA

**Keywords:** lung cancer, immune cells, lung stromal cells, CD73, adenosine, ecto-nucleotidase

## Abstract

**Simple Summary:**

CD73 and adenosine are garnering significant attention in lung cancer research. CD73, an enzyme crucial for adenosine production, is found in various cells, including immune cells, within our lungs. During stress or cancer, adenosine levels increase from their usual low levels. Notably, lung tumor cells, particularly non-small cell lung cancer, express substantial CD73. The CD73 expression in the resident lung cells and immune cells may set an environment prone to cancer development, underscoring the importance of understanding cell interactions governing tumoral growth and behavior. Presently, the treatment with immune checkpoint inhibitors aims to help our body fight cancer; however, they do not work for every patient. Researchers propose targeting CD73 and adenosine to enhance these treatments to work better. CD73 acts as a “stop” signal for immune cells fighting cancer, a role immune checkpoint inhibitors aim to reverse. Blocking CD73 might amplify treatment effectiveness. Beyond this, CD73 shapes immune cell interactions and activity against cancer. Deciphering CD73 and adenosine’s roles in lung cancer may potentially reshape the therapeutic landscape and offer novel therapeutic opportunities.

**Abstract:**

CD73 and adenosine have gained prominence in lung cancer research. The *NT5E* gene encodes CD73, known as an ectonucleotidase, which plays a crucial role within tumor cells, with immune-suppressive properties. Beyond cancer, CD73 exerts an influence on cardiac, neural, and renal functions, affecting cardiac, neural, and renal functions. CD73’s significance lies in its production of extracellular adenosine. It is notably expressed across diverse cell types within the immune and stromal lung microenvironment. CD73 expression amplifies in lung tumors, especially non-small cell lung cancer (NSCLC), often aligned with key oncogenic drivers like mutant *EGFR* and *KRAS*. CD73/adenosine pathway seems to be involved in tumoral immunoevasion, hampering the use of the immune checkpoint inhibitor (ICI) and correlating with therapy resistance. Despite the partial success of current ICI therapies, the CD73/adenosine pathway offers promise in enhancing their effectiveness. This comprehensive review explores recent insights into lung cancer’s CD73/adenosine pathway. It explores roles within tumor cells, the lung’s stromal environment, and the immune system. Ranging from pre-clinical models to clinical trials, potential therapies targeting the adenosine pathway for lung cancer treatment are discussed below.

## 1. Introduction

In the last years, CD73/adenosine has been prompted as a novel target in lung cancer research. CD73 is encoded by the gene *NT5E* and is crucial in numerous tumor cell-intrinsic and extrinsic functions [1]. Till now, CD73 is a dimeric ecto-5-nucleotidase (5′-NT) that is expressed on the exterior side of the plasma membrane. Each subunit has N- and C-terminal domains resembling bacterial 5′-NT enzymes. The metal ion binding site is in the N-terminal domain, while the substrate binding site and dimerization interface are in the C-terminal domain. The active enzyme site, formed by residues from both domains, is between them [2].

CD73 was studied in cancer for its role in immune suppression, but recent studies have elucidated far more functions related to this molecule [1,3,4]. CD73/adenosine signaling has been described to be involved in cardiac function [5,6], neural signaling [7,8], and renal function [9,10], among other defined functions [11].

CD73 is the primary manufacturer of extracellular adenosine. ATP is released from the cell, and CD39 at the cell surface dephosphorylates ATP to AMP. Subsequently, in a second step, CD73 converts AMP to adenosine, CD73 on the cell surface of cells is the rate-limiting step in the generation of extracellular adenosine [12]. A non-canonical pathway also leads to AMP production, but both paths eventually converge to CD73 activity [13]. Adenosine is a nucleoside necessary for cellular functions and for building RNA and DNA. In homeostatic conditions, the extracellular adenosine concentrations within tissues are in the low nM rank; however, cellular stress and cancer formation concentrations trigger the production of adenosine that can reach up to the 100 µM range [14,15,16].

CD73 is widely expressed in different tissues since adenosine is generated by hypoxia and chronic inflammation to limit tissue injury and host inflammatory damage [17]. CD73 transcription factors are principally regulated by signal transducers and activators of transcription 3 (STAT3)/hypoxia-inducible factor-1α (HIF-1α) cascade. Many cytokines, including transforming growth factor-β (TGFβ), interferons (IFNs), tumor necrosis factor (TNFα), interleukin-1β (IL-1β), and prostaglandin E2 (PGE2), induce CD73 expression.

**Figure 1:**
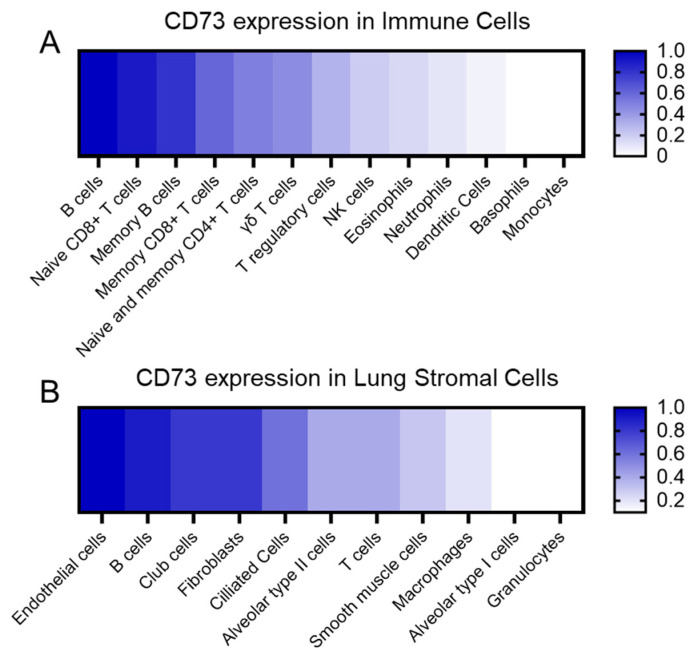
CD73 relative expression. (**A**) CD73 expression in the immune cell populations relative to the B cells, which are the main immune cells expressing CD73. (**B**) CD73 expression in the lung stromal cells relative to endothelial cells, which are the main expressing population. We summarized information from public transcriptomic and proteomic databases.

CD73 is highly expressed in several cell types within the lung (Figure 1). The main expressing cells are the endothelial cells, fibroblasts, and different subsets of epithelial cells such as club cells, ciliated cells, and with less intensity, alveolar type II cells. Immune cells, including naïve B cells, naïve CD8-T cells, and memory B cells, are significant expressers of CD73, while CD4-T cells and lung resident macrophages express it to a lesser extent, according to immGen and Human Protein Atlas data. Lung tumors, mainly non-small cell lung cancer (NSCLC), showed robust CD73 expression signatures among all cancer types, particularly when associated with common oncogenic drivers of NSCLC, such as mutant epidermal growth factor receptor (EGFR) and KRAS [18,19]. CD73-derived adenosine binds to the surface of various immune cells such as the regulatory (Foxp3^+^) T cell (Tregs), effector T cell, natural killer (NK) cell, myeloid-derived suppressor cell (MDSC), macrophages, and B cell. It can regulate the tumor microenvironment and the tumor cell immunoevasion capacity.

Nowadays, immune checkpoint inhibitor (ICI) therapies benefit only a subset of patients, challenging the scientific community to identify responders even with the existing predictive biomarkers like PD-L1. CD73 and the adenosine pathway are well known to disrupt the cytotoxic function of T cells, which is currently the main target of most clinical agents, and play a key role in tumor immunogenicity. Combining ICI drugs with adenosine pathway inhibitors holds promise for lung cancer therapy, despite the association of increased CD73 with therapy resistance [18,20,21].

This review provides insights into the latest literature on the adenosine pathway, particularly CD73, in lung cancer and other tumors. We will highlight the signaling of the CD73/adenosine pathway in tumoral cells and their interaction in lung stroma and immune system players. We will explain the current knowledge of this pathway in both pre-clinical models and clinical settings focusing on lung cancer. Finally, the review summarizes existing evidence of therapies and clinical trials involving the adenosine pathway, discussing prospects for treating lung cancer.

## 2. Materials and Methods

### 2.1. Search Strategy and Study Selection

The review was conducted under the Preferred Reporting Items for Systematic Reviews and Meta-Analyses (PRISMA) guidelines [22]. The literature search was independently conducted by three researchers (M.S., O.M-C., and R.G-P.), using the two major databases, PubMed and Scopus, before March 2023. The search used different combinations of terms such as cancer AND (CD73) or lung cancer AND (CD73). In addition, CD73 AND (tumor microenvironment OR lung stromal cells OR immune cells).

Articles were considered eligible for the review if they met the following inclusion criteria: (i) evaluation of the biological effect of the signaling through CD73/adenosine pathway in all kinds of cancer processes or lung cell populations, (ii) studies in vitro and in vivo with data of CD73 in tumoral cells or lung cell populations, (iii) data related to cell proliferation, wound healing, cell invasion, hypoxia, cellular pathways related to migration and invasion processes that CD73 and adenosine were mentioned. Exclusion criteria were (1) case reports, editorials, abstracts, unpublished studies, book chapters, and commentaries; (2) in silico analysis only; (3) full-length articles in non-English language; and (4) none of the above-mentioned assays.

Articles without free full text available were searched through the University Autonoma de Barcelona digital library, or via direct contact with the authors. The full-text versions of all potentially relevant studies were screened, and disagreements were resolved through discussions between the authors until consensus was achieved. A search of references of included studies and previous reviews on the topic was also performed by hand to include additional relevant studies according to our selection criteria.

### 2.2. Systematic Review Process and Data Extraction

Overall, around 2000 articles were preliminarily identified in the literature search. After excluding duplicates, three independent reviewers (M.S., O.M-C., and R.G-P.) screened titles and abstracts of 956 records. Investigators were blinded to each other’s decisions. Information about study design and methodology, cells or subjects evaluated, reported results, and effects were extracted and summarized by each author. In the end, 125 articles were eligible due to their scientific interest and used for this review.

## 3. CD73 in the Interaction between Tumor Cells and Immune and Lung Resident Cells

In this section, we describe the interaction between tumor cells and the immune cells and stromal cells in the lung tissue focusing on the CD73/adenosine axis. Stromal–immune cell crosstalk essentially modifies the lung microenvironment, which may promote a carcinogenic milieu and may impact tumoral cell proliferation.

### 3.1. Cell–Cell Interaction between Tumor Cells with Immune Cells

**Figure 2:**
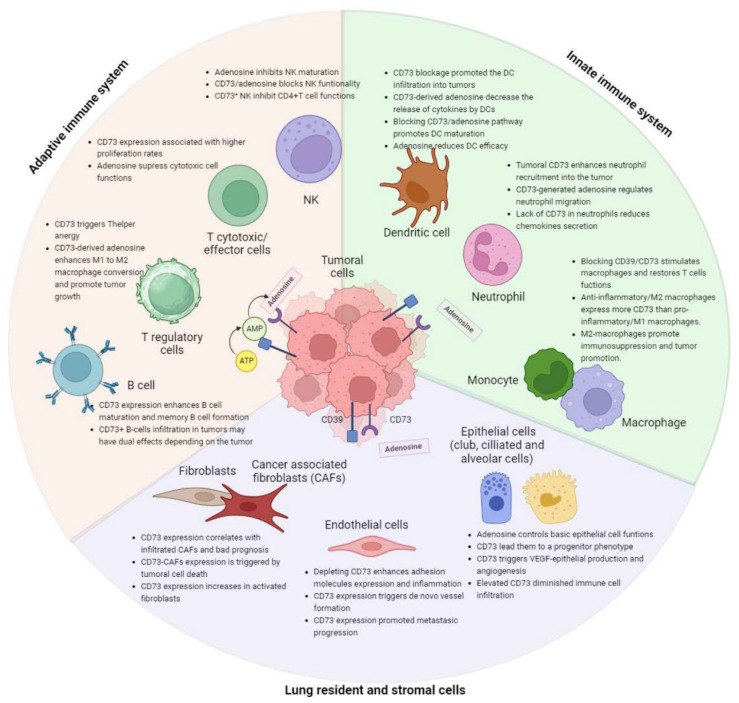
Summary of the effects of CD73/adenosine within all lung resident and immune cells related to carcinogenesis.

The interaction between tumor cells and immune cells is a heterogeneous and complex process that plays a fundamental role in the development and progression of cancer. While the immune system has mechanisms like NK cells, cytotoxic T cells, and antigen-presenting cells to eliminate cancer cells, their interaction can lead to factors promoting tumor growth. Tumor cells, in turn, develop mechanisms to inhibit immune responses. One of the molecules identified as an important regulator of this interaction is the CD73 receptor. CD73 overexpression is associated with cancer progression, suppression of the immune system, poorer prognosis, and increased metastasis risk in various cancers [23]. The CD39/CD73 complex and the extracellular adenosine play a crucial role in cancer cells evading the immune system. This chapter will explore how CD73/adenosine expression impacts various immune cells and influences the lung tumoral microenvironment; we have summarized this chapter in a diagram in Figure 2.

#### 3.1.1. T Cells

The CD39/CD73 complex critically inhibits the activation and expansion of tumor-specific T cells, hindering the eradication of cancer cells. Morandi et al. found that CD73 significantly impedes T cell proliferation in human melanoma lines [24]. Research by Chalmin et al. demonstrated that IL-6 and TGFβ-induced Th17 cells expressing CD73 led to adenosine generation, suppressing CD4+ and CD8+ T cell functions [25]. Elevated CD73 levels on Treg cells in cancer patients contribute to Treg cell differentiation and long-term anergy, hindering effector T cell functions through adenosine production [26,27]. Lower CD73 expression on effector T cells has been shown to enhance their antitumor function [28]. CD73 also orchestrates lymphocyte and monocyte trafficking via its adhesion role [29,30]. Furthermore, it contributes to the suppression of Treg and Th17 cells by the generation of adenosine in the cellular environment, which enhances the conversion of proinflammatory M1 macrophages to reparative M2 macrophages, which promotes tumor growth [31].

In melanoma, CD73 high tumors exhibit fewer infiltrating CD8+ T cells and CD56+ NK cells infiltration [32]. In vitro, primary human melanoma cell lines suppress T cell proliferation through the CD73/adenosine pathway [24]. In human head and neck cancer, the soluble CD73 in peripheric blood and the CD73 expression on peripheral T cells correlate with CD73 expression on CD8+ T cells in tumors [33]. CD8+ CD73+ T cells mediate immunosuppression, while CD8+ CD73− T cells exhibit exhaustion features, being less proliferative [33]. CD8 CD73− T cells exhibit features of exhaustion revealed by high expression of inhibitory receptors such as PD-1 and TIGIT, reduced capacity of cytokine production, and high rates of apoptosis [34]. CD73-derived adenosine by Tregs has been proposed as a resistance mechanism to anti-PD-1 therapy in murine tumor models. Therapeutically expanded human Treg cells are highly suppressive due to HIF1A-triggered expression of CD73 [35]. CD73 defined a subset of effector CD4+ T cells (Teffs) enriched in polyfunctional Th1.17 cells. CD39+ Tregs selectively target CD73+ Teffs, and they were functionally blunted in breast and ovarian tumors [36]. The absence of inhibitory immune checkpoints in tumor-infiltrating CD73+ Teffs suggests that CD73 could be specifically chosen in response to the pressure exerted by immune checkpoint blockade therapy. This implies that CD73 might serve as a unique and nonredundant target for the restoration of antitumor immunity.

#### 3.1.2. B Cells

B cells are the main immune cell subset expressing CD73, among B cells, the memory B cell subset is the most expressers of CD73 [21]. The regulation of CD73 expression in B cell responses appears to be precise, with a gradual increase observed in germinal center (GC) B cells post-immunization. CD73 is prominently expressed in T follicular helper cells interacting with GC-B cells but is absent among plasma cells (PC) and plasmablasts (PB) [37].

Thompson et al. noted that CD73 expression correlates with a memory state, with low levels in neonatal B cells, increased levels in infant B cells before IgG responses, and exceptionally high CD73 expression in IgG-switched B cells in the tonsil [1,38,39]. The upregulation of CD73 in antigen-experienced [40] and GC B cells [41] supports the concept that CD73 function is associated with memory B cell formation. However, the specific role of CD73 in humoral responses remains poorly understood. The increased function of the CD39/CD73/adenosine pathway has been associated with B cell hyperactivation in patients with chronic hepatitis [42], and with IgM and granzyme B production during malaria infection [43], which supports the fact that CD73 is regulated in B cells depending on pathological events.

When analyzing T-dependent B cell response, no differences in the formation of GCs, memory B cells, plasmablasts, and PC between wild-type or CD73 knockout mice were found [37]. Nevertheless, a significant reduction in bone marrow PC was observed in mice lacking CD73, indicating the necessity of CD73 expression on bone marrow-derived cells for optimal PC responses. Deletion of CD73 from either B or T lymphocytes replicated these findings, suggesting that CD73 expression is sufficient on one of the two cell types to promote B cell differentiation and that observations are consistent with its function as an ectoenzyme. Overall, CD73-dependent adenosine signaling is crucial in the mature GCs and essential for establishing the long-lived PC compartment, unveiling a novel role for CD73 in humoral immunity. In colorectal cancer (CRC), CD73 is highly expressed on tumor-infiltrating B cells, particularly on class-switched memory B cells, and is absent on plasmablasts. CD73+ B cell infiltration in CRC tumors correlates with improved overall survival, while metastasized patients have fewer tumor-infiltrating CD73+ B cells. Neoadjuvant therapy reduces CD73+ B cell numbers and CD73 expression in CRC tumors [44]. Miller et al. demonstrated that the inhibition of CD73 using a specific antibody stimulates CD73+ B cells, promoting antigen-specific immune responses with protective immune characteristics, and inducing B cell redistribution in lymphoid tissues in cancer patients [45].

In melanoma patients, a significant increase in circulating CD49b^+^CD73^+^ B cells was observed. These patients also had higher amounts of tissue-infiltrating IgG4^+^ CD49b^+^ CD73^+^ B cells expressing proangiogenic cytokines and linked to a bad prognosis [46].

B cell content in the tumor microenvironment (TME) of classic Hodgkin lymphoma (HL) is known to be associated with prognosis. Grund et al. detected downregulation of CD73 in Hodgkin lymphoma with low B cells and suggested that the immunosuppressive milieu provoked by CD73/adenosine is affecting B cells’ functionality [47].

In chronic lymphocytic leukemia (CLL), pathological B cells expressed significantly lower levels of CD73 than normal B cells. The patients with higher levels of CD73 correlated with a poor clinical and biological prognostic factor, and presented a shorter overall survival, supporting the idea that CD73 may play a role in CLL pathophysiology, and may be of potential value as a prognostic marker and therapeutic target [48].

#### 3.1.3. Natural Killers (NKs)

Under normal conditions, NK cells express low levels of CD73, but its expression can increase particularly in situations like gastrointestinal stromal tumors. Adenosine, produced by CD73, hampers NK cell maturation under hypoxic conditions. A2AR activation by CD73-generated adenosine inhibits NK cell functions. Tumor-infiltrating NK cells exhibit increased CD73 expression, and the frequency of CD73^+^ NK cells is associated with larger tumors in breast cancer patients. CD73^+^ NK cells suppress CD4+ T cell proliferation and cytokine production compared to CD73^-^ NK cells [49].

Inhibiting CD73 enhances T lymphocyte infiltration and tumor cell killing, proposing a strategy to boost antitumor immunity by modulating CD73/adenosine signaling in NK cells, potentially mitigating tumor progression in cancer patients.

#### 3.1.4. Dendritic Cells (DCs)

CD73 is lowly expressed in DCs, but CD73 function in the tumoral microenvironment induces changes in DC cell behavior. CD73 blockage in a preclinical lung cancer mouse model increased DC infiltration and decreased cytokine release by DCs [50,51].

The immunosuppressive mediators, such as adenosine, in the tumor microenvironment, compromise the efficacy of DC-based cancer immunotherapy. Using a selective A2A adenosine receptor antagonist and CD73 inhibitor in combination improved the outcomes of DC-based cancer vaccination, reducing tumor growth, prolonging mouse survival, and enhancing specific antitumor immune responses [52].

Blocking the CD73/adenosine pathway promoted DC maturation and immune cell infiltration, and decreased the risk of CRC lung metastasis. Lin et al. have shown that tumor CD73 expression inhibited the recruitment of immune cells and correlated with a poor prognosis in colon adenocarcinoma patients [53]. Targeting the cancer-derived adenosine through the CD73 pathway emerges as a promising strategy to enhance the effectiveness of DC-based cancer immunotherapy.

#### 3.1.5. Neutrophils

Neutrophils co-express CD39/CD73, along with all four adenosine receptors, allowing them to generate and receive adenosine in an autocrine feedback loop [54]. Unstimulated neutrophils continuously produce and secrete a low amount of adenosine, and in an inflammatory microenvironment, activated neutrophils become a major source of tissue adenosine [55,56]. CD73 presence in the tumor microenvironment is associated with immune cell infiltration, including neutrophils [57].

Dysregulation of the CD39/CD73 axis can lead to an uncontrolled increase in neutrophil activation [58], and CD73-generated adenosine regulates neutrophil migration [59]. Hence, the lack of CD73 in neutrophils could directly impede their migration into inflamed tissues, while CD73 knockout mice show an accumulation of neutrophils in the lung in response to inhaled lipopolysaccharide (LPS) [60]. The absence of CD73 in neutrophils reduces chemokine secretion, protecting against excessive activation of neutrophils, and reducing airway inflammation [61].

Neutrophils in tumors can exhibit either an anti-tumorigenic or a pro-tumorigenic effect [62,63]. At early stages of tumor expansion, neutrophils are almost exclusively at the periphery of the tumor, but at later stages, neutrophils can also be found distributed among the tumor cells [64]. The content of neutrophils is associated with the lymphocyte content and a poorer response to PD(L)1 therapy in NSCLC [65]. While there is limited evidence on CD73/adenosine and neutrophils in cancer studies, targeting neutrophils is worth considering, as their presence has been linked to enhanced tumor cell survival and metastatic potential in various mouse models [66,67,68].

#### 3.1.6. Monocytes/Macrophages

CD39 is the main ectonucleotidase expressed in monocytes/macrophages, with less than 5% of human healthy CD14+ monocytes expressing CD73. However, resident tissue macrophages can express both enzymes [69]. Zanin et al. demonstrated that human pro-inflammatory macrophages (M1) exhibit lower CD39 and CD73 levels than anti-inflammatory macrophages (M2) [70]. Macrophages regulate inflammation through ATP catabolism, while adenosine impacts macrophage function by promoting an anti-inflammatory activation and increasing IL-10-induced STAT-3 production [71].

In the tumor microenvironment, monocytes differentiate into M2-like macrophages called tumor-associated macrophages (TAMs), promoting immunosuppression and tumor progression. CD73 expression on cancer cells generates adenosine, reinforcing immunosuppression by TAMs and contributing to tumor immune evasion. TAMs expressing CD39/CD73 suppress CD4+ T cell proliferation through adenosine generation [72]. Antibodies against CD39/CD73 promoted antitumor immunity by stimulating macrophages and restoring T cell activation in cancer patients [73].

### 3.2. Cell–Cell Interaction between Tumor Cells with Lung Resident Cells

CD73 is widely expressed on the surface of numerous resident lung cell types, stomal and immune cells, including endothelial cells, and airway epithelial cells [74]. Understanding adenosine’s role in lung parenchyma and alveoli requires identifying its sources during homeostasis. Adenosine may originate from the interstitial compartment and penetrate the epithelial airway cells to reach the lumen. Transcriptomic and protein data indicated that lung endothelial cells are the main expressing cells in the lung, followed by club cells, fibroblasts, ciliated cells, alveolar cells, and smooth muscle cells (Figure 1). This chapter reviews the expression and function of CD73 in the key resident lung cells to elucidate its role in maintaining pulmonary homeostasis, and it is summarized in a schematic way in Figure 2.

#### 3.2.1. Epithelial Cells

CD73 expression is not homogeneous across all epithelial cells in various tissues; for instance, in the pancreas [75], breast [76], and the gastrointestinal tract [77] there is differential expression of CD73 across epithelial cell compartments. In the lung, CD73 is localized to the mucosal surface of columnar epithelial cells, particularly on the apical plasma membrane and underlying basal cells [78]. CD73 is responsible for the production of adenosine on the mucosal surface of human airway epithelial cells and plays a major role in the regulation of adenosine-mediated epithelial functions [74]. Extracellular adenosine controls epithelial functions such as mucociliary clearance, an essential airway defense mechanism against bacterial infection. The novel epithelial EpCAM^+^CD73^+^ cell population shows progenitor properties [79] and can rise to a pseudostratified epithelium in a 2D air–liquid interface and generate lung organoids with alveolar-like features recapitulating a mucociliary–secretory cell fate found in vivo. So, CD73 may be considered a progenitor-like marker in epithelial cells.

In early stage endometrial carcinoma, CD73-generated adenosine protects epithelial integrity by a physiological response to protect epithelial integrity, and its loss allows tumor progression [80]. In a preclinical melanoma model, endothelial CD73 promotes the production and release of VEGF, encourages endothelial cell proliferation and migration, and supports the formation of new tumor blood vessels [81].

In bladder cancer, CD73-negative epithelial cells are significantly associated with poor survival, although CD73 expression in stromal fibroblasts or lymphocytes had no predictive power [82]. In prostate cancer, Leclerc et al. reported that elevated CD73 levels in the normal adjacent prostate epithelium are linked to a shorter period of biochemical recurrence-free survival, while elevated CD73 levels in the tumor stroma were associated with a prolonged duration of biochemical recurrence-free survival [83].

Observations by Wang et al. revealed that reduced CD73 expression in the blood vessels of glioma patients, in comparison to the levels in the normal brain, could potentially compromise the blood–brain barrier. This scenario might pave the way for favorable conditions that facilitate tumor growth [84].

In triple-negative breast cancer, there was a notable adverse relationship between the expression of CD73 on epithelial tumor cells and the infiltration of CD45+ immune cells. Notably, patients exhibiting both elevated CD73 epithelial expression and diminished CD45+ immune cell infiltration experienced the poorest survival outcomes [20].

#### 3.2.2. Endothelial Cells

CD73 is expressed abundantly on endothelial cells [85], and its depletion in HUVEC cells induces a pro-inflammatory phenotype comparable to TNF-α stimulation [86]. CD73 depletion increased surface levels of ICAM-1, VCAM-1, and E-selectin, along with increased translocation of NF-κB to the nucleus. This effect is mimicked by changes in adenosine levels [87,88]. CD73-depleted HUVEC cells become elongated, which increases stress fibers and endothelial permeability, indicating CD73′s role in suppressing pro-inflammatory response in human endothelial cells.

In vivo, acute inflammation, driven by TNF-α, reduces endothelial CD73 activity [89], and interferon-beta-1a (IFN-β1a) increases CD73 synthesis and diminishes vascular leakage [90]. CD73 expression is linked to a decrease in adhesion molecules, and that effect is due to the production of adenosine. CD73 knockout mice exhibit elevated vascular expression of VCAM-1 and ICAM-1, increased NF-κB, and heightened vascular leakage in response to hypoxia [91,92]. Inhibiting endothelial CD73 induces neutrophil accumulation in the lungs following LPS treatment, suggesting that CD73/adenosine protects against neutrophil recruitment [60]. In vitro, the silencing of CD73 with a siRNA enhanced monocyte and neutrophil adhesion to endothelial cells [93].

The importance of CD73 in endothelial cell function is evident in CD73-deficient mice, emphasizing its role in maintaining vascular barrier homeostasis. The dysregulation of the endothelial barrier function is associated with various diseases, including cancer [94]. Leukocyte adhesion into the endothelial cell inhibits CD73 activity, reducing local adenosine production favoring vascular permeability and leukocyte transmigration [95].

CD73 expression on tumor cells and endothelial cells contributes to enhancing tumor angiogenesis. In a breast cancer model, anti-CD73 antibody treatment reduces tumor VEGF levels and angiogenesis. CD73 expression on non-hematopoietic cells, likely endothelial cells, promotes metastatic progression [96,97]. CD73’s multifaceted impact on endothelial cells underscores its significance in inflammation regulation, leukocyte trafficking, and tumor angiogenesis.

#### 3.2.3. Fibroblasts

The critical source of CD73 activity in the tumor microenvironment remains unspecified, as numerous cell types express it. Notably, fibroblasts and myofibroblasts express CD73 and are able to produce adenosine, playing significant roles in the tumor microenvironment. CD73 expression correlates positively with the degree of infiltration of cancer-associated fibroblasts (CAFs) and endothelial cells in most cancers [98]. Interestingly, CD73 has been used as one of the CAF markers in several studies [99]. It has been demonstrated that cancer-associated fibroblasts (CAFs) constitute the prominent CD73^hi^ population, particularly in human CRCs. Clinically, high CAF abundance in CRC tissues strongly correlates with elevated CD73 activity and poor prognosis [100]. CAFs actively influence the immune landscape in the tumor microenvironment by regulating extracellular matrix production and secreting chemokines and growth factors that can affect cancer cell function [101,102]. Tumoral cell death triggers the CAF–CD73 expression, enhancing the CD73 immune checkpoint. Another interesting observation is that CD73 gene expression increases during myofibroblastic differentiation, which may be important in patients developing cancer and presenting a fibrotic chronic respiratory disease [103]; in this context, CD73 expression in myofibroblasts on those lungs may promote carcinogenesis.

## 4. Gene Signatures Regulating Immune Pathways in Tumor Microenvironment (TME)

The dynamic interplay between tumor and immune cells undergoes changes throughout carcinogenesis, endowing tumor cells with the capability to evade the host immune system [104]. Briefly, the prolonged selective pressure exerted by the immune system to eliminate cancer cells prompts the emergence of tumor clones adept at evading immune-mediated destruction, thereby promoting tumor growth [105]. These strategies hinge on the physiological mechanisms of immune response suppression and are the basis for the development and design of novel immunotherapeutics, as discussed below.

### 4.1. Gene Signatures Favoring Immune Response: IFNγ-Signature

IFNγ is a soluble cytokine primarily produced by the T-lymphocytes and natural killer cells in response to various inflammatory or immune stimuli. It binds to its receptors IFNGR1 and IFNGR2, activating Janus kinases (JAK1 and JAK2) and the signal transducer and activator of transcription 1 (STAT1) [106]. This activation prompts the expression of numerous immune-related genes, including those involved in immunorecognition, antigen presentation (*TAP1*, *TAP2*, *B2M*), and immune checkpoints like PD-L1 [107].

Genetic defects in IFNGR1/2 or JAK1 and JAK2 promote tumor refractoriness to IFNγ [108], particularly noted in melanoma where these alterations have been identified and associated with resistance to treatment with ICIs [109,110]. In lung cancer, limited research has revealed *JAK1* and *JAK2* loss-of-function mutations, with *JAK2* mutant cells found to be unresponsive to IFNγ treatment [111]. Moreover, following antigen presentation to the T cell lymphocytes and IFNγ release, the expression of co-inhibitory immune checkpoints, such as cytotoxic T-lymphocyte-associated antigen 4 (CTLA-4), the programmed cell death 1 (PD-1) or the PD-1 ligand (PD-L1) molecules occurs, a mechanism known as adaptive immune resistance. Most current immune checkpoint inhibitors in solid tumors target CTLA-4, PD-1, and PD-L1, serving as monoclonal antibodies that hinder their functions, while numerous other compounds are undergoing clinical investigation [112,113].

Thus, a proficient IFNγ signaling pathway and the presence of an inflammatory gene signature enriched by IFNγ response genes are emerging as a predictive biomarker for ICI with prognostic implications [114]. Indeed, Cristescu and colleagues proposed an 18-gene T-cell-inflamed gene expression profile, which was consistently associated with the response to immunotherapy in various types of cancer, including lung cancer [115].

While an association between CD73 and PD-L1 expression has been observed [17], it remains uncertain whether CD73 exerts an influence on the IFNγ pathway.

### 4.2. Gene Signatures Associated with an Immunosuppressive Milieu: The Adenosine Signature

The hypoxic tumor microenvironment induces hypoxia-inducible factor 1α (HIF-1 α), leading to the upregulation of adenosinergic molecules, including CD39, CD73, and the adenosine receptor A2BR [34,116]. These molecules are associated with metastatic disease and adverse clinical outcomes across various tumor types, including lung cancer [12,117].

Hypoxia induced the release of extracellular ATP within the tumor microenvironment, subsequently converted to adenosine, via CD39 and CD73. This process initiates a potent anti-inflammatory response, inhibiting the function of multiple host cells within TME, such as mast cells, endothelial cells, macrophages, neutrophils, NKs, DCs, and lymphocytes. Adenosine’s impact includes the stimulation of T cell anergy and the differentiation of CD4 cells into Foxp3^+^ Tregs, contributing to tumors’ immune escape and cancer growth [23,118,119,120].

Additionally, TGF-β enhances Treg cell function, suppressing T cytotoxic cells and NK cells [121]. It also drives differentiation of myeloid-derived suppressor cells into protumorgenic terminally differentiated MDSC characterized by high levels of cell surface CD39/CD73 expression [122].

Cristescu et al. identified a stroma/epithelial-to-mesenchymal transition/TGF-β signature, that was negatively associated with immunotherapy response and predictors of resistance, and may be potential targets for combination therapy strategies [115].

## 5. Novel Therapeutics Affecting Adenosine Pathway within TME

Efforts to counter adenosinergic molecules as a therapeutic approach have seen considerable progress. Preclinical studies using adenosine receptor inhibition in various tumor models have shown promising results, including restored immune cell function and tumor regression. For example, concurrent inhibition of A2AR and CD73 with monoclonal antibodies demonstrated synergistic effects compared with monotherapy in a mouse model with metastatic disease [123]. Another therapeutic strategy using MEDI9447 (MedImmune), a potent selective anti-CD73 human monoclonal antibody, either alone or in combination with the anti-PDL1 durvalumab, has been evaluated in phase 1 clinical trial with several tumor types, including lung cancer. Encouraging signs of antitumor activity were noted in tumor types typically resistant to immunotherapy [124]. CPI-444 (Corvus Pharmaceuticals), an orally selective A2AR antagonist, restored T cell signaling, and cytokine production suppressed by adenosine analogs in vitro. It showed promising outcomes in preclinical studies, including complete responses in more than half of the mice and induction of memory T cell responses, and with a synergistic activity in combination with anti-PD-L1 or anti-CTLA [125].

Given the broad activity of anti-PD(L)1 inhibitor but with some limitations, the blockage of the adenosine signaling pathway represents a novel therapeutic approach that has demonstrated an optimal safety profile and enhanced overall response rates in early phase trials, particularly using CD73 and A2AR inhibitors. In Table 1 we show a comprehensive presentation of drugs targeting adenosinergic molecules that are at the moment used in clinical trials to improve the outcome for lung cancer patients. Beneficial outcomes for both monotherapy and combinations have been mostly lower than expected based on preclinical studies, emphasizing the need for more precise patient selection or biomarker integration to predict and optimize patient responses.

## 6. Conclusions

In summary, CD73’s multifaceted roles, from immune suppression to its pivotal role in generating extracellular adenosine, highlight its emerging significance in lung cancer and broader implications in cancer biology.

Abundantly expressed in various lung cell types, CD73 overexpression is strongly linked to non-small cell lung cancer (NSCLC) and common oncogenic drivers. Its interactions with immune cells, including Tregs, NK cells, and MDSCs, shape the tumor microenvironment, influencing immunoevasion. In the evolving landscape of cancer therapy, the combination of ICIs with adenosine pathway inhibitors presents a promising avenue. However, challenges such as therapy resistance associated with increased CD73 expression need to be addressed. This review provides a comprehensive overview of the current literature on the CD73/adenosine pathway in lung cancer emphasizing intricate signaling networks involving CD73 in tumoral cells, stroma, and the immune system. Additionally, it has explored the current state of therapies and clinical trials targeting the adenosine pathway, underscoring the need for more precise patient selection and biomarker integration to optimize outcomes.

In conclusion, CD73’s expanding role in cancer biology, particularly in lung cancer, offers a promising avenue for future research and therapeutic development. Understanding the nuances of the CD73/adenosine axis and its intricate interactions within the tumor microenvironment holds great potential for improving the treatment landscape for lung cancer patients.

## Figures and Tables

**Table 1:** Overview of clinical trials investigating targets within the adenosine signaling pathway in lung cancer.

Molecular Target	Drug Combinations	Study Population	Outcomes Reported	ClinicalTrial.gov Identifier (Accessed on 4 October 2023)
CD73	CPI-006 (mupadolimab) +/− ciforadenant +/− pembrolizumab	Advanced cancers (including NSCLC)	NA	Phase 1/1b: NCT03454451
	Oleclumab (MEDI9447) + Osimertinib	EGFR mut NSCLC	100% TEAEs (any)	Phase 1b/2: NCT03381274 (arm A)
	Oleclumab + Durvalumab	EGFR wt NSCLC	ORR 38.3% mDoR 12.9m	Phase 2: NCT03822351 (COAST)
	BMS-986179 (BMS) + Nivolumab	Advanced cancers (including NSCLC)	NA	Phase 1/2a: NCT02754141
	Oleclumab + Osimertinib or +AZD4635(A2A agonist)	EGFR mut NSCLC	NA	Phase 1/2: NCT03381274
	Oleclumab +/− Durvalumab	Advanced solid tumors including EGFR mut NSCLC	NA	Phase 1: NCT02503774
	PT119 +/− Anti-PD-1	Advanced cancers (including NSCLC)	NA	Phase 1: NCT05431270
	LY3475070 +/− Pembrolizumab	Advanced cancers (including NSCLC)	NA	Phase 1: NCT04148937
	Sym024 +/− Sym021 (antiPD1)	Advanced cancers (including NSCLC)	NA	Phase 1: NCT04672434
	TJ004309 + Atezolizumab (antiPDL1)	Advanced cancers (including NSCLC)	NA	Phase 2: NCT03835949
A2AR	Imaradenant (AZD4635) + oleclumab	EGFR mut NSCLC	80% TEAEs (any)	Phase 1b/2: NCT03381274 (arm B)
	PBF-509 + PDR001 (antiPD1)	Advanced NSCLC	NA	Phase I/Ib: NCT02403193
	NIR178 + PDR001	Advanced cancers (including NSCLC)	Terminated (sponsor decision)	Phase 1: NCT03549000

NSCLC: Non-small cell lung cancer, TEAEs: treatment-emergent adverse events, NA: Non-applicable, mut: mutant, wt: wild-type.
